# Right atrial cardiac rhabdomyoma with premature foramen ovale restriction: A case report

**DOI:** 10.3892/ol.2014.2605

**Published:** 2014-10-10

**Authors:** YI-DAN LI, YI-HUA HE, ZHI-AN LI, PING WEI

**Affiliations:** 1 Department of Ultrasound, Beijing Chaoyang Hospital, Capital Medical University, Beijing 100020, P.R. China; 2 Department of Ultrasound, Beijing Anzhen Hospital, Capital Medical University, Beijing 100029, P.R. China; 3 Department of Pathology, Beijing Chaoyang Hospital, Capital Medical University, Beijing 100020, P.R. China

**Keywords:** atrial cardiac rhabdomyoma, intrauterine fetal mortality, foramen ovale restriction, postnatal tuberous sclerosis complex, fetal echocardiography, tomographic ultrasound imaging

## Abstract

Fetal cardiac rhabdomyoma is the most common cardiac tumor in fetuses. However, this benign tumor can cause hemodynamic repercussions and intrauterine fetal mortality. The present study reports a case of rare fetal cardiac rhabdomyoma located in the right atrium, accompanied by premature restriction of the foramen ovale and moderate pericardial effusion, as determined by tomographic ultrasound imaging (TUI). Fetal mortality subsequently occurred late in the second trimester of pregnancy and the diagnosis was confirmed by pathology. The present study discusses the occurrence and diagnosis of this rare abnormality. TUI mode with spatio-temporal image correlation offline imaging provides the physician with clear views of abnormal intracardiac structures in the beating heart. With improvements in sonographic technology, the diagnosis of fetal cardiac rhabdomyoma may be easier and more accurate in the clinical arena.

## Introduction

Fetal cardiac rhabdomyoma is a rare condition, however, it is the most common cardiac tumor in fetuses, accounting for 60–86% of primary fetal cardiac tumors (1). Fetal cardiac rhabdomyoma is most often diagnosed by fetal echocardiography. Cardiac rhabdomyomas have a tendency for spontaneous regression, however, they may cause arrhythmias, hemodynamically significant obstruction, heart failure and sudden mortality (2). Surgery is not usually required, as cardiac rhabdomyoma often regress and thus, may be managed conservatively and monitored by serial echocardiograms and electrocardiograms, with the exception of cases in which location leads to hemodynamic compromise or untreatable arrhythmias (3). The present study reports a rare case of fetal cardiac rhabdomyoma located in the right atrium, accompanied by premature restriction of the foramen ovale and moderate pericardial effusion, as determined by tomographic ultrasound imaging (TUI) and confirmed by pathology. Fetal mortality occurred late in the second trimester of pregnancy. Written informed consent was obtained from the patient.

## Case report

**Figure 1:**
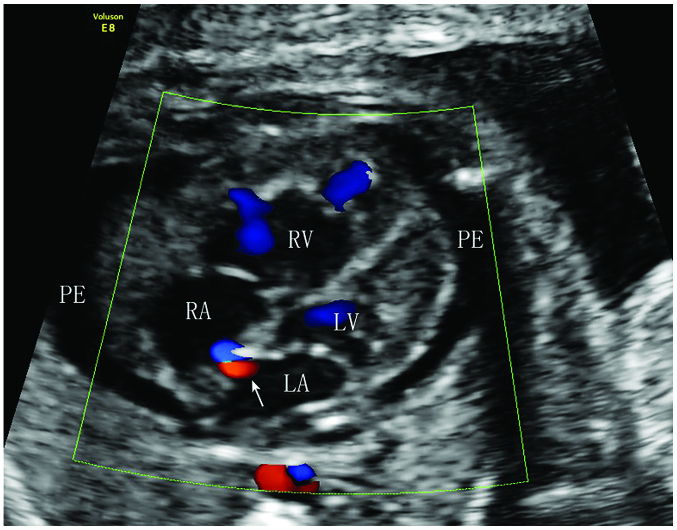
Color Doppler imaging indicating an extremely narrow foramen ovale flow (indicated by the white arrow). PE, pericardial effusion; RA, right atrium; RV, right ventricle; LV, left ventricle; LA, left atrium.

**Figure 2:**
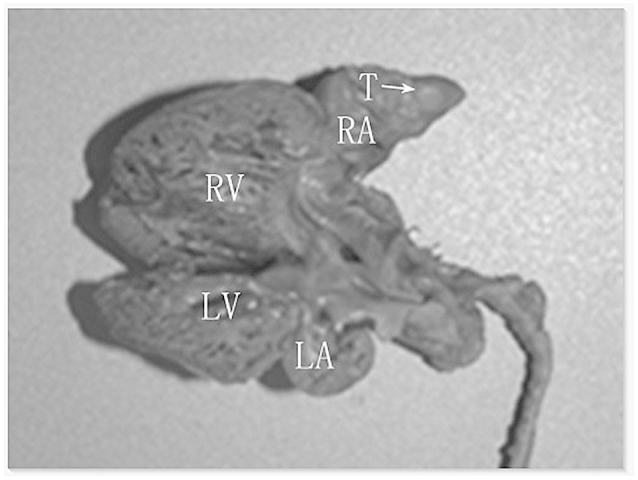
Autopsy revealed a single tumor mass (arrow) in the wall of the right atrium. The tumor measured 4.3×4.0 mm in size. T, tumor; RA, right atrium; RV, right ventricle; LV, left ventricle; LA, left atrium.

**Figure 3:**
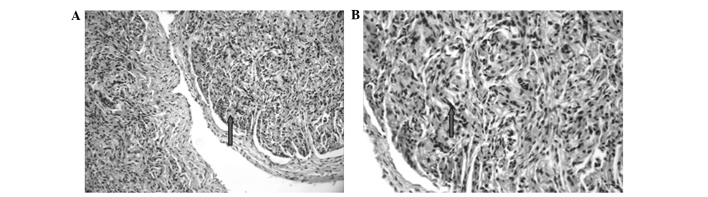
Hematoxylin and eosin (H&E) staining. The image under a light microscope revealed evidence of nodular hyperplasia with clear boundaries, swirl-like cells, cords and a random orientation. The cells possessed a strong eosinophilic cytoplasm, with a slightly increased nuclear size and chromatin condensation (arrows). However, atypia was not remarkable. H&E, original magnification (A) ×200 and (B) ×400.

**Figure 4:**
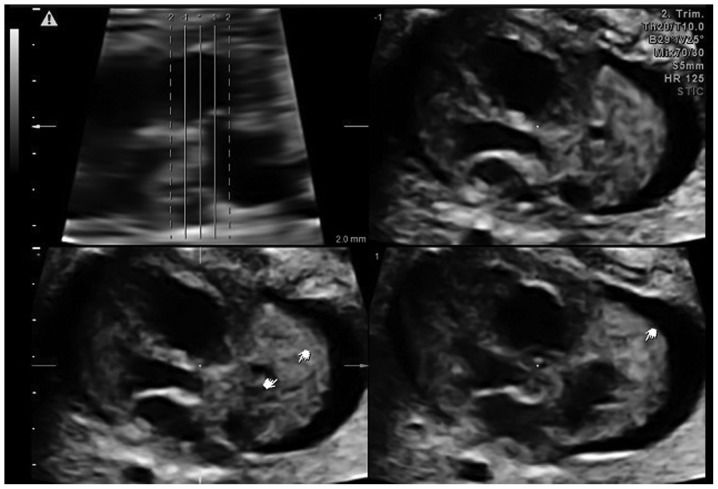
Tomographic ultrasound imaging results demonstrating a solitary tumor (indicated by pointers) located in the right atrium near the right atrial appendage (lower panels).

A 30-year-old female, gravid 2 para 0, was referred to the Department of Ultrasound, Beijing Anzhen Hospital (Beijing, China), following a routine prenatal ultrasound examination at Cangzhou City Maternal and Child Care Service Centre (Cangzhou City, China) where pericardial effusion (PE) was detected. The patient had previously suffered a spontaneous abortion at 11 weeks of pregnancy two years ago and the reasons for this remained unclear. Two-dimensional (2D) and three-dimensional (3D) ultrasound imaging was performed using the Voluson E8 ultrasound system (4–8 MHz probe; GE Healthcare, Cleveland, OH, USA). The evaluation demonstrated a single live intrauterine pregnancy of 26 weeks. The fetal echocardiography four-chamber view assessment demonstrated a 6.3-mm thickening of the right atrial wall. Moderate PE was also observed. Color Doppler imaging indicated a narrow foramen ovale flow of only 1.9 mm in diameter (Fig. 1). A bicaval view revealed superior vena cava and inferior vena cava diameters of 3.0 mm and 3.4 mm, respectively. The patient was informed of the possibility of fetal abnormalities and asked to attend weekly follow-ups. However, the patient did not feel quickening two days later and fetal mortality was diagnosed by fetal echocardiography. An autopsy of the fetus revealed that the heart was slightly enlarged, with a subendocardial nodule of 4.3×4.0 mm in size located in the right atrium. The nodule was sharply demarcated, exhibiting a reddish-gray color with a moderately firm texture and the typical appearance of a rhabdomyoma (Fig. 2). Histological hematoxylin and eosin staining revealed nodular hyperplasia with clear boundaries, swirl-like cells, cords and a random orientation. The tumor cells possessed a strong eosinophilic cytoplasm, with slightly increased nuclear size and chromatin condensation. However, atypia was not evident (Fig. 3A and B). The images were reviewed offline by tomographic ultrasound imaging (TUI) and spatio-temporal image correlation (STIC) imaging, which clearly displayed a 5.0×4.0-mm mass located in the right atrial wall area (Fig. 4). No major or minor manifestations of tuberous sclerosis or other notable family histories were documented.

## Discussion

Fetal cardiac rhabdomyoma is the most common cardiac tumor in fetuses, with an extremely low reported incidence rate in fetal echocardiograms (0.17%) and accounting for 60–86% of primary fetal cardiac tumors. Currently, fetal echocardiography is the primary tool for the early detection of primary cardiac rhabdomyoma (4). However, in the present case, only right atrial wall thickening was observed, as the tumor was not noticeable in traditional 2D imaging. A possible mass was observed following offline review of the images using TUI. In echocardiography, rhabdomyomas are often observed as round, homogeneous and hyperechogenic ventricular masses, occasionally appearing in the ventricles and septal wall areas of the atrium (5). The occurrence of a single tumor in the right atrium and masses in the pericardium are rare (6). Although fetal cardiac rhabdomyomas are histologically benign, the increased size and hydrops are significantly associated with poor neonatal outcomes. In certain cases, these features may cause intrauterine mortality. Overall, the risk of fetal mortality is 4–6% (7).

The incidence of the premature restriction of foramen ovale-accompanied tumors in the right atrium is rare. Premature foramen ovale restriction can lead to pericardial or pleural effusion, right-sided heart failure, dysrhythmia, congestion, non-immune hydrops and ascites. Fetal mortality may be associated with premature restriction of the foramen ovale. Also, up to 80% of prenatally-diagnosed cardiac rhabdomyomas have been associated with postnatal tuberous sclerosis complex (TSC) (8). However, in the present case, the parents had no family history of TSC.

Other cardiac tumors, including fibromas, teratomas, hemangiomas and myxomas, are extremely rare (9). Fibromas differ from cardiac rhabdomyoma, usually originate from the left ventricular apex and are mostly solitary. Teratomas are extracardiac, with attachment to the aortic root or pulmonary artery, and tend to grow within the pericardial cavity. Myxomas are located in the atrium and have a stalk that allows free movement during the cardiac cycle, and hemangiomas are usually situated at the base of the heart adjacent to the atria (10).

The ultrasound images in the present study suggested a solid, non-calcified tumor located in the right atrium. The differential diagnosis for this finding may include a rhabdomyoma, teratoma or hemangioma, among other diagnoses. Although fetal cardiac hemangiomas and teratomas are also often found in the right atrium, hemangiomas demonstrate a more complex echogenicity, with cystic and solid parts mixed with calcifications (11), while teratomas can be either cystic or solid. The differential morphological characterization of cardiac tumors requires fetal cardiac evaluation. As the use of echocardiography to differentiate rhabdomyoma from teratoma or hemangioma for a single cardiac mass located in the atrium is occasionally difficult, the combination of other newly-developed prenatal imaging techniques, including STIC, physician experience, family history and patient symptoms, may be more appropriate for the confirmation of the diagnosis (12). In the present study, it was hypothesized that reviewing images offline by TUI mode with STIC could reveal a solitary tumor located in the right atrium.

DeVore *et al* first reported the prenatal diagnosis of a cardiac tumor in 1982 (13). The prenatal diagnosis of fetal cardiac tumors has become feasible due to the advancement in fetal echocardiography. TUI and STIC are two novel types of volume data imaging techniques that are processed by 3D and 4D ultrasound. STIC associated with the TUI mode is a novel modality, which allows for the exhibition of a complete sequential analysis of cardiac structures on a single panel that demonstrates all echocardiographic transverse views at the same time. The fetal cardiac rhabdomyoma diameter range has previously been reported to be 4–52 mm in the majority of cases (14), which is consistent with the present results, as the tumor size was 5.0×4.0 mm. However, it is difficult to detect tumors of this size using the conventional 2D mode; initially, only a thick fetal right atrial wall was identified in the present study. By contrast, the tumor was clearly observed by TUI and STIC. STIC is a technique that allows examination of the fetal heart within a real-time 3D volume, through display in a cine-loop. At the same time, real-time 3D echocardiography with instantaneous volume rendering reveals mobile views of cardiac tumors (15). Through surface rendering of the STIC volume, an operator has the ability to observe virtual planes that are impossible to observe by conventional 2D ultrasound imaging. Thus, the 3D/4D ultrasound modalities may have advantages at evaluating certain abnormalities in the fetal cardiovascular structure (16).

In summary, the present study reports a case of rare fetal cardiac rhabdomyoma located in the right atrium, accompanied by premature restriction of the foramen ovale and moderate pericardial effusion, which led to fetal mortality late in the second trimester of pregnancy. It was demonstrated that TUI mode with STIC offline imaging provides the physician with clear views of abnormal intracardiac structures of the beating heart. With improved sonographic technology, the diagnosis of fetal cardiac rhabdomyoma may become easier and more accurate in the clinical arena.
